# Electrochemical Sensor for Hydrogen Peroxide Based on Prussian Blue Electrochemically Deposited at the TiO_2_-ZrO_2_–Doped Carbon Nanotube Glassy Carbon-Modified Electrode

**DOI:** 10.3389/fchem.2022.884050

**Published:** 2022-07-05

**Authors:** Lenys Fernández, Jocelyne Alvarez-Paguay, Gema González, Rafael Uribe, Diego Bolaños-Mendez, José Luis Piñeiros, Luis Celi, Patricio J. Espinoza-Montero

**Affiliations:** ^1^ Escuela De Ciencias Químicas, Pontificia Universidad Católica Del Ecuador, Quito, Ecuador; ^2^ Yachay Tech University, School of Physical Sciences and Nanotechnology, Urcuqui, Ecuador; ^3^ Instituto Venezolano De Investigaciones Científicas, Centro De Ingeniería Materiales y Nanotecnología, Caracas, Venezuela; ^4^ Departamento De Ingeniería Química, Escuela Politécnica Nacional, Quito, Ecuador; ^5^ Departamento De Física, Escuela Politécnica Nacional, Quito, Ecuador

**Keywords:** carbon nanotubes, titania nanoparticles, zirconia nanoparticles, Prussian blue, electrochemical sensors

## Abstract

In this investigation, a hydrogen peroxide (H_2_O_2_) electrochemical sensor was evaluated. Prussian blue (PB) was electrodeposited at a glassy carbon (GC) electrode modified with titanium dioxide– and zirconia-doped functionalized carbon nanotubes (TiO_2_.ZrO_2_-fCNTs), obtaining the PB/TiO_2_.ZrO_2_-fCNTs/GC-modified electrode. The morphology and structure of the nanostructured material TiO_2_.ZrO_2_-fCNTs was characterized by transmission electron microscopy, the specific surface area was determined *via* Brunauer–Emmett–Teller, X-ray diffraction, thermogravimetric analysis, and Fourier transform infrared spectroscopy. The electrochemical properties were studied by cyclic voltammetry and chronoamperometry. Titania-zirconia nanoparticles (5.0 ± 2.0 nm) with an amorphous structure were directly synthesized on the fCNT walls, aged during periods of 20 days, obtaining a well-dispersed distribution with a high surface area. The results indicated that the TiO_2_.ZrO_2_-fCNT–nanostructured material exhibits good electrochemical properties and could be tunable by enhancing the modification conditions and method of synthesis. Covering of the nanotubes with TiO_2_-ZrO_2_ nanoparticles is one of the main factors that affected immobilization and sensitivity of the electrochemical biosensor. The electrode modified with TiO_2_-ZrO_2_ nanoparticles with the 20-day aging time was superior regarding its reversibility, electric communication, and high sensitivity and improves the immobilization of the PB at the electrode. The fabricated sensor was used in the detection of H_2_O_2_ in whey milk samples, presenting a linear relationship from 100 to 1,000 μmol L^−1^ between H_2_O_2_ concentration and the peak current, with a quantification limit (LQ) of 59.78 μmol L^−1^ and a detection limit (LD) of 17.93 μmol L^−1^.

## 1 Introduction

A sensor is a device that allows the transformation of a physicochemical process into an analytical signal, through a recognition element that connects the analyte with the transducer, showing an electrical signal (Yan et al., 2015). The proper design of a sensor is of utmost importance so that the results of any analysis are accurate and at the same time reliable. Therefore, nanotechnology, electrochemistry, and analytical chemistry have been combined developing devices with diagnostic capacity (Srinivasan et al., 2015).

Electrochemical sensors take advantage of the redox characteristic of chemical species for the development and application of analytical techniques. Electrochemical techniques are very attractive because they do not generate a high cost, have a fast response, and are easy to integrate into complex investigations.

For an electrochemical sensor to have a good performance, the recognition element must be correctly immobilized on its surface, where the design and development of simple and compatible materials are extremely important. In order to reach the latter, organic (Li et al., 2016) and inorganic nanomaterials are properly combined to obtain the optimum efficiency. This is an emerging area in nanotechnology.

Carbon nanotubes (CNTs) have been the subject of much research due to their unique and remarkable mechanical, electrical, thermal, and elastic properties. CNTs have a relevant application in nanodevices, particularly in sensors, where they are used to increase analytical performance, when they are functionalized with suitable materials and through interconnection and transduction processes on their surface.

For a good performance of the CNT as part of an electrochemical sensor, its surface must be chemically modified (Montes et al., 2016). Metal oxide nanoparticles bound to CNT surfaces have received notable interest due to their applications in the design of functional nanostructures in electrochemistry that include high adsorption capacity, catalytic properties, biocompatibility, and suitable surface sizes that favor the efficient interaction of the analyte on the sensor (Solanki et al., 2011; Srinivasan et al., 2015; Montes et al., 2016).

Prussian blue (PB) is an inorganic compound widely used to modify electrodes for electrochemical sensors. The most important feature of PB when it comes to analytical applications is its electrocatalytic activity for the reduction of hydrogen peroxide (H_2_O_2_) and oxygen (Itaya et al., 1984). Electrodeposition of PB under certain conditions has been reported to lead to the synthesis of a selective electrocatalyst to reduce H_2_O_2_ in the presence of oxygen (Karyakin et al., 1996). Due to its high activity and selectivity, which are commonly the properties of biological catalysts, PB has been called artificial peroxidase (Itaya et al., 1984). PB as the H_2_O_2_ transducer is very advantageous (Karyakin et al., 2004) compared to the platinum electrode (the electrocatalyst part that has been regarded as the benchmark for H_2_O_2_ and O_2_), and PB-modified electrodes are three orders of magnitude more active and selective in the reduction and oxidation of H_2_O_2_ in neutral media in the presence of oxygen (Karyakin et al., 1996).

The monitoring of low levels of H_2_O_2_ is of great importance for modern medicine, environmental control, and various branches of industry (Gutiérrez et al., 2011; Lv et al., 2020; Shu and Tang, 2020; Wang et al., 2020; Zhang et al., 2020; Hou et al., 2021; Xu et al., 2021). In particular, in biological applications, for example, there is the presence of H_2_O_2_ in the human body that is converted into OH radicals with high reactivity; an overproduction of OH and O_2_ at cell openings promote cell damage and tissue malfunctioning (Rhee et al., 2010; Grisham, 2013). Similarly, the detection of H_2_O_2_ is a diagnostic response of medical devices such as glucose sensors, which are in the presence of oxygen, where H_2_O_2_ is produced by the action of glucose oxidase (Sajjadi Kouchesfehani et al., 2017). They also play an essential role in everyday life, such as monitoring food, bleaching processes, and even regulating human metabolic processes. Therefore, the detection of H_2_O_2_ is of relevant importance.

The combination of PB with CNTs and nanostructured metal oxides (Roushani et al., 2013a; Roushani et al., 2013b; Salimi et al., 2013; Roushani and Karami, 2014; Roushani and Dizajdizi, 2015; Roushani and Dizajdizi, 2016) can generate novel results for good electrical communication between the recognition system and the electrode surface. Zirconium oxide (ZrO_2_) nanoparticles have been used as an electrochemical biosensor platform for oral cancer detection by cyclic voltammetry (Zong et al., 2007; Zhao et al., 2020; Rathee et al., 2021). A nanostructure material from ZrO_2_ nanoparticles on CNTs was used to immobilize myoglobin showing excellent electrocatalytic activity for the reduction of H_2_O_2_ (Yang et al., 2020)^.^ On the other hand, titanium dioxide (TiO_2_) has a large surface area and unique electronic and chemical properties (Zhang et al., 2011; Bai and Zhou, 2014; Liu et al., 2017; Guerrero et al., 2019). Considering its electronic band structure, TiO_2_ is rich in electrons and belongs to the category of n-type semiconductors. TiO_2_ nanomaterials are used for sensors in the detection of organic compounds that are soluble in aqueous media. Furthermore, they are used for the detection of gases and biological and chemical substances. Consequently, it may be stated that research on electrochemical sensors indicates that the immobilization of the recognition system is a task that must be fulfilled in order to produce an electrical connection between the analyte and the electrode. The modified CNTs facilitate the detection of a good interconnection path, and this would lead to a better detection of H_2_O_2_ that has an improved response in the analysis (Li et al., 20182018).

The development of catalysts for H_2_O_2_ reduction is of major interest for electrochemical sensors. PB is a cheap, efficient, and robust catalyst for H_2_O_2_ reduction, whose simple preparation allows the development of a series of H_2_O_2_ sensors on the nanometer scale with the advantage to miniaturize operational devices and promising to probe in real time the production of H_2_O_2_ in living cells by using nanoelectrodes. In this context, the present work presents the development of an electrochemical H_2_O_2_ sensor based on a glassy carbon (GC) electrode modified with carbon nanotubes functionalized (fCNTs) with a mixture of nanostructured TiO_2_ and ZrO_2_, using PB immobilized on the electrode surface as artificial peroxidase.

An electrochemical biosensor based on PB electrodeposited at a GC electrode modified with TiO_2_.ZrO_2_-fCNTs for the detection of H_2_O_2_ has been reported for the first time here. The effect of covering the CNT walls with TiO_2_-ZrO_2_ nanoparticles is effective on the immobilization of PB, and the sensitivity of the electrochemical biosensor is studied.

## 2 Materials and Methods

### 2.1 Reagents and Instrumentation

All solutions were prepared with distilled/deionized water (18 MΩ resistivity, Darmstadt, Germany). Carbon nanotubes were obtained from NANOCYL®NC7000(Austin, TX, USA). Nitric acid (HNO_3_, 69.2 wt%) and hydrogen peroxide ( $H_{2}O_{2}$ 30% v/v) were purchased from Sigma-Aldrich (Darmstadt, Germany). Potassium phosphate monobasic ( $KH_{2}PO_{4}$ ) and sodium hydroxide (NaOH, 99.9% p/p) were purchased from Fisher Scientific (Waltham, MA, USA). Phosphate-buﬀered saline (PBS: $KH_{2}PO_{4}$ + $K_{2}\mathrm{HP}O_{4}$ + KCl, at different pH values) was used as a supporting electrolyte. Potassium ferricyanide ( $K_{3}\left[\mathrm{Fe}{\left(\mathrm{CN}\right)}_{6}\right]$ ), iron trichloride hexahydrated ( $\mathrm{FeC}I_{3}\cdot 6H_{2}O$ ), and potassium chloride (KCl) were obtained from BDH Chemicals (Philadelphia, PA, USA); hydrochloric acid (HCl, 37%) was from Fisher Scientific (Waltham, MA, USA); glassy carbon (GC, Φ=3mm , geometric area = 0.0706 cm^2^), silver/silver chloride reference electrode (Ag/AgCl), and graphite rod counter electrode were from CH Instruments (Austin, TX, USA); sulfuric acid ( $H_{2}SO_{4}$ , 98%) was from Fisher Scientific (Waltham, MA, USA); 1 μm, 0.3 µm, and 0.05 µm alumina powder were from CH Instruments (Austin, TX, USA); dimethylformamide (DMF) was from BDH Chemicals (Philadelphia, PA, USA); and poly (diallyldimethylammonium chloride) (PDDA, 4% w/w in water) was from Sigma.

### 2.2 Functionalization of Carbon Nanotubes

Functionalization of CNTs was carried out in order to insert functional groups on the CNT walls that allow the chemical interaction with other species. CNTs (NANOCYL®NC7000, Austin, TX, USA) of 90% purity were used, with an average diameter of 9.5 nm, transition metal content <1%, and surface area 250 m^2^/g resistivity 10^–4^ Ωm. A previous pre-functionalization was carried out on the pristine CNTs using 3 mol L^−1^ HNO_3_ and 1 mol L^−1^ H_2_SO_4_, under reflux at 80°C with constant agitation of 400 rpm for 6 h. The CNTs were washed, filtered, and dried (16 h) and finally gently crushed in a mortar and sieved. Then, the latter CNTs were functionalized employing an acid mixture HNO_3_–H_2_SO_4_ (1:3) under reflux at 80°C for 30 min. Then, the functionalized multiwalled carbon nanotubes (fCNT) were washed with deionized water until neutral pH was reached and dried at 80°C for 10 h.

### 2.3 Synthesis of the fCNT/TiO_2_.ZrO_2_–Nanostructured System

The nanostructured materials, fCNTs/TiO_2_, fCNTs/ZrO_2_, and fCNTs/TiO_2_.ZrO_2_, were obtained by *in situ* sol-gel synthesis of the metallic oxides (MO_2_) on the fCNTs. The synthesis is based on that reported by Gonzalez et al. (2012). Table 1 shows the number of reactants used. In summary, the following procedure was followed: a solution of titanium isopropoxide (Ti(OPri)_4_) or/and zirconium isopropoxide (Zr(OPri)_4_) was prepared in 100 ml of isopropanol. This solution was gradually added to the fCNTs that were suspended in 100 ml of isopropanol under continuous agitation and left under reaction for 12 h. In addition, two different concentrations of fCNTs with respect to the metal oxides (MO_2_) were used (36 and 100 w/w). The suspension was allowed to stand for 1–20 days at room temperature, the solvent was removed, and the material was dried under vacuum at 80°C for 4 h. The materials were calcined at two different temperatures 80 and 500°C for 2 h under argon atmosphere. The final product was ground to obtain a fine powder.

**TABLE 1:** Amount of reactants used in the synthesis of the nanostructured materials fCNT/MO_2_(c)T.t.

Reactant	Nanostructural systems	
	fCNTs/TiO_2_.ZrO_2_ (100) T.t (*)	fCNTs/TiO_2_ZrO_2_ (36)T.t (*)
fCNT(g)	0.1500	0.080
Titanium isopropoxide T (mL)	0.3	0.3
Zirconium isopropoxide (mL)	0.4	0.4
Isopropanol (mL)	10.3	11.5
Propanol (mL)	–	–
Deionized wáter (mL)	0.14	0.16
Acetic acid (mL)	0.17	0.19

(*) (100) and (36) are wt% of CNTs wrt the metallic oxide, T is calcination temperature (80 or 500°C), and t is aging time (1 or 20 days).

### 2.4 Characterization of the Nanomaterials

Transmission electron micrographs (TEMs) were taken using a JEOL 1220 microscope operating at an accelerating voltage of 100 kV. The samples were prepared by the wet suspension technique in an ethanol/water solution (70% v/v), and a drop of the suspension was placed on a TEM carbon–coated Cu grid.

Infrared spectra were acquired using a Nicolet iS10 FTIR spectrometer (Peabody, MA USA). The samples were prepared in KBr pellets. The frequency range was from 4,000 to 400 cm^−1^ with 64 scans at a resolution of 2 cm^−1^.

X-ray diffraction patterns were obtained in a SIEMENS D5005 diffractometer (Radeberg, Germany) with a wavelength of 1.54178 Å in the range of 10°–80° 2θ degrees with a speed of 0.02/0.52 s.

Zeta potential was carried out for all the synthesized nanomaterials in Zetasizer Malvern Pananalytical equipment. Measurements were performed on an aqueous suspension of carbon nanotube samples in distilled water as solvent. The environmental conditions of the analysis were a temperature of 24.1°C and a humidity of 45.7%. The samples were subjected to five measurement runs. From these data, the mean and standard deviation values of the zeta potential were calculated.

Differential scanning calorimetry and thermogravimetry analyses (DSC-TGA) were performed on a TA instrument SD Q600 ((TA Instrument, New Castle, DE, USA) from room temperature to 1,000°C, with a heating rate of 10°C/min under an air atmosphere.

The specific surface area was determined using the Brunauer–Emmett–Teller (BET) method in ASAP 2010 Micromeritics equipment (Micromeritics, Norcross (Atlanta, GA, USA). The samples were previously degassed under vacuum at 150°C for 4h, and the desorption isotherms were taken at liquid nitrogen temperature (77 K).

### 2.5 Glassy Carbon Electrode Modification

#### 2.5.1 Cleaning of the Glassy Carbon Electrode

Mechanical polishing of the electrode was carried out with 0.5 and 0.03 µm alumina powder during 5 min, followed by an electrochemical cleaning using a potential from −1.2 to −1.6 V with a scan rate of 100 mV s^−1^ for 30 cycles.

#### 2.5.2 Electrode Preparation of fCNTs/TiO_2_.ZrO_2_(c) T.t/GC

A suspension of fCNT/TiO_2_.ZrO_2_(c) T.t in DMF was prepared at a concentration of 5.0 mg mL^−1^ and sonicated during 1 h. This suspension was kept at 20°C. To modify the GC electrode, 10 µL of the fCNT/TiO_2_.ZrO_2_ suspension was added dropwise on its surface and was allowed to dry under infrared radiation for 20 min before use. In addition, electrodes of fCNTs/TiO_2_(c)T.t**/**GC and fCNTs/ZrO_2_ **(c)**T.t/GC were prepared following an identical procedure.

#### 2.5.3 Electrode Preparation of PB-fCNTs/TiO_2_.ZrO_2_ (c)T.t /GC

On the fCNTs/TiO_2_.ZrO_2_(c)T.t/GC electrode, 10 µL of the PDDA solution was pipetted and left to dry for 15 min at 50°C; subsequently the PB was electrodeposited on this surface. The electrodeposition of PB was achieved in an aqueous solution containing 2.5 mmol L^−1^ K_3_ [Fe(CN)_6_] + 2.5 mmol L^−1^ FeCl_3_.6H_2_O + 0.2 mol L^−1^ KCl + 0.2 mol^−1^ HCl, at a potential of +0.4 V for 240 s. Later, the PB-fCNTs/TiO_2_.ZrO_2_(c)T.t/GC electrode was activated by cycling at a potential range of −0.2 to 1.2 V at a sweep speed of 50 mV s^−1^ in 0.2 mol L^−1^ KCl + 0.2 mol L^−1^ HCl for eight cycles. The PB-fCNT/TiO_2_.ZrO_2_(c)T.t/GC electrode was left to dry at 50°C for 15 min, and then, a 10 µL volume of PDDA solution was dropped onto the modified electrode and dried for 15 min at 50°C.

### 2.6 Electrochemical Characterization of the Modified Electrodes

Experiments for the electrochemical characterization of the modified electrodes were carried out by cyclic voltammetry (CV) in a 0.1 mol L^−1^ PBS, in a range of a scan rate of 10–120 mV s^−1^. Using the Randles–Sevick equation (Ec.1), the diagnosis of the electrochemical process on the modified electrodes was carried out:

$$i_{p}=2.69x10^{5}n^{3/2}A\;C\;D^{1/2}v^{1/2}$$ (1)

where i_p_ (A) is the peak current, A is the electrode area in cm^2^, C is the concentration of the species in solution (mol cm^−3^), *v* is the potential scan rate (V s^−1^), and D (cm^2^ s^−1^) is the diffusion coefficient.

Electrochemical capacitance of each modified electrode was obtained by CV in 0.1 mol L^−1^ PBS (pH 3.0) solution and at potentials of 0.20–0.55 V. According to the following equation (Eq. 2), we obtain

j=IcA=Cdlv, (2)

where C_dl_ (F) is the double layer capacitance, I_c_ is the current, and j (A cm^−2^) is the current density.

Surface concentration of PB on the electrodes was calculated using the following equation (Eq. 3):

$$I_{P}=\frac{n^{2}F^{2}Αv\Gamma C}{4RT},$$ (3)

where n is the number of electrons transferred in the redox process, F is the Faraday’s constant, R (J mol^−1^ K^−1^) is the gas constant, T (K) is the temperature, A is the electrode area in cm^2^, and *v* is the potential scan rate (V s^−1^).

### 2.7 Hydrogen Peroxide Detection

Prior to obtention of the calibration plot for the quantification of H_2_O_2_, diagnostics tests were made through CV in order to determinate the response signal of the analyte in 0.1 mol L^−1^ PBS (pH 3.0) in a potential range from −0.40 to +0.60 V and a scan rate of 40 mV s^−1^.

To evaluate the sensitivity and linearity of the response to H_2_O_2_ at the PB-fCNTs/TiO_2_.ZrO_2_(c)Tt/GC-modified electrode, chronoamperometric measurements were made in PBS, pH 3.0, with continuous stirring and adding aliquots of 0.05 mol L^−1^ H_2_O_2_ solution every 30 s to obtain a calibration plot from 150 to 1.028 μmol L^−1^. The experiments were carried out keeping the temperature at 20°C ± 2.

#### 2.7.1 Evaluation of the Determination of Hydrogen Peroxide in the Presence of Interferents

The selectivity of the PB-fCNTs/TiO_2_.ZrO_2_(c)T.t/GC electrode was evaluated by comparing the amperometric response of H_2_O_2_ before and after adding possible interferences in a PBS (pH 3.0). Ascorbic acids (15 mmol L^−1^), glucose (15 mmol L^−1^), and dopamine (20 mmol L^−1^) were used as interferents; subsequently, chronoamperometric measurements were made in PBS (pH 3.0), by adding aliquots of 0.05 mol L^−1^ H_2_O_2_ every 20 s.

#### 2.7.2 Evaluation of the Determination of Hydrogen Peroxide in Real Samples

For the application in a practical analysis, the PB-fCNTs/TiO_2_.ZrO_2_(c)T.t/GC electrode was used, and recovery percentages (R%) were calculated in fortified whey milk samples with different concentrations of H_2_O_2_ (250 μmol L^−1^ and 600 μmol L^−1^).

## 3 Results and Discussion

### 3.1 Characterization of the Nanostructured Materials

#### 3.1.1 Fourier Transformed Infrared Spectroscopy

**FIGURE 1:**
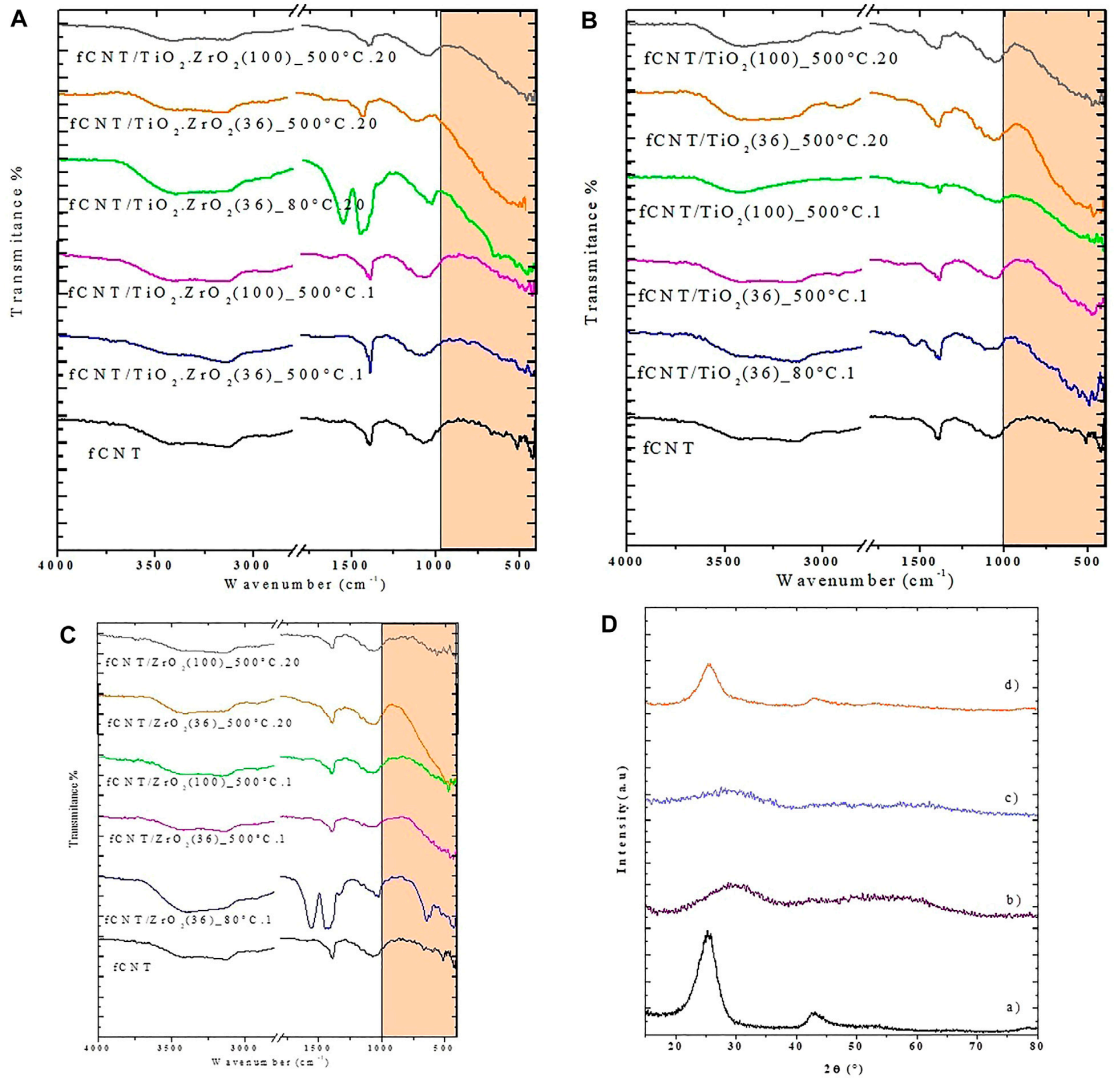
FTIR spectra of the nanostructured materials obtained under different synthesis conditions: **(A)** fCNT/TiO_2_ZrO_2_; **(B)** fCNT/TiO_2_; **(C)** fCNT/ZrO_2_; and **(D)** XRD patterns of a) fCNT, b) ZrO_2_ 80°C, c) TiO_2_- 80°C. and d) fCNT/TiO_2_(36) 80. °C.1.

The FTIR spectra for the different synthesized materials are presented in Figure 1. Compared to the spectrum of the fCNTs (Figures 1A–C, black line spectrum), no great differences are observed for the materials aged for 1 day, suggesting that the titania and zirconia particles have not been formed yet or are too small. On the contrary, significant differences are observed for the materials aged for 20 days, especially in the region between 400 and 950 cm^−1^. The materials fCNT/TiO_2_.ZrO_2_(36)500°C.20 and fCNT/TiO_2_.ZrO_2_(100)500°C.20 show an intense band in this region associated with Ti-O-Ti, Zr-O-Zr or Ti-O-Zr bonding. This band is also observed for the fCNT/TiO_2_ and fCNT/ZrO_2_ materials, suggesting a chemical interaction between the metal oxides and the fCNT walls. This suggests that the bonding for the fCNT/TiO_2_.ZrO_2_ material could be Ti-O-Zr. This broad band is located between that of fCNT/TiO_2_ and fCNT/ZrO_2_ (Figures 1A–C, spectrum in green and orange lines). The materials calcined at 80°C present bands similar to those found for uncalcined zirconia.

#### 3.1.2 X-Ray Diffraction

**FIGURE 2:**
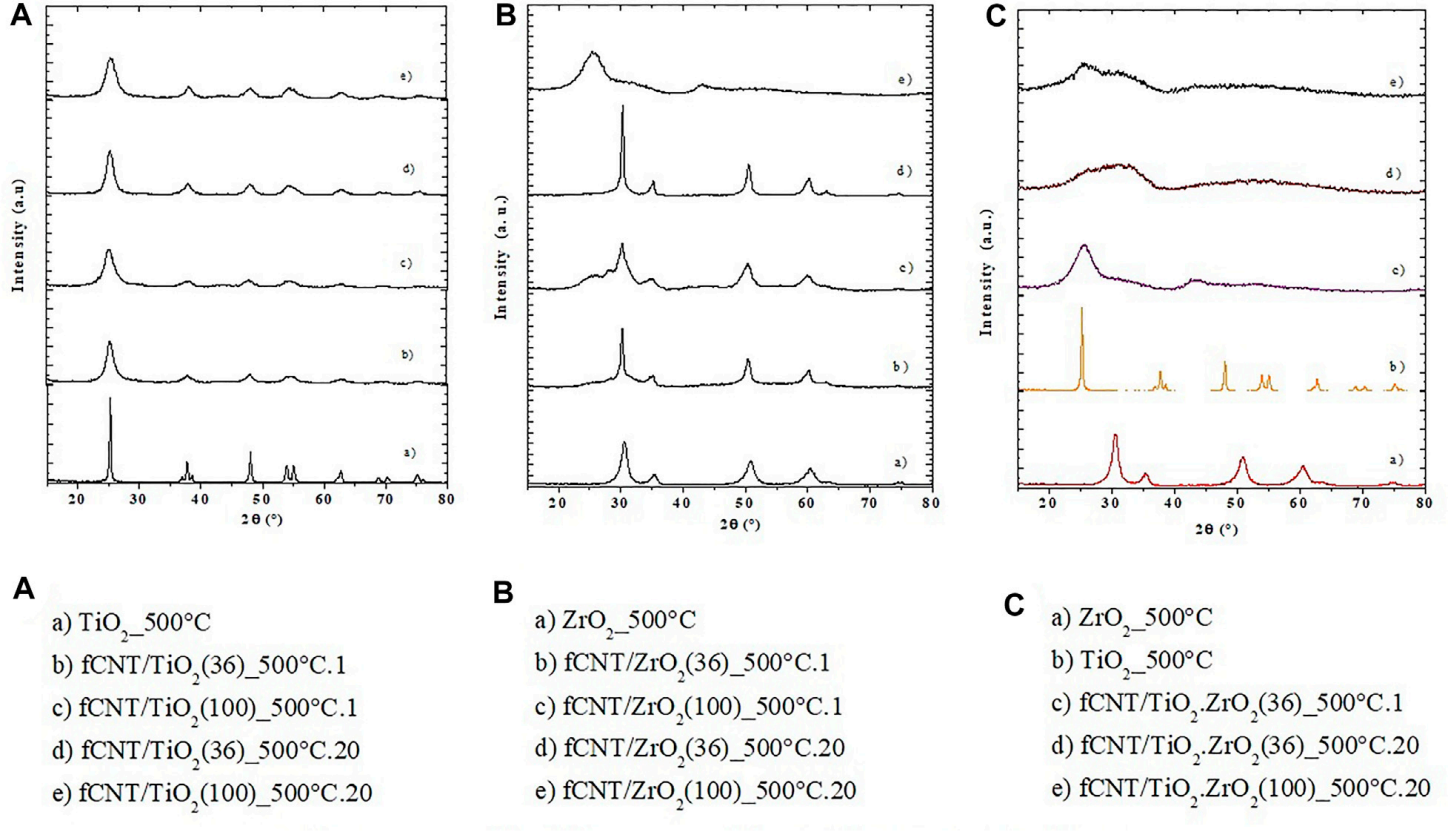
XRD patterns of the different materials: **(A)** TiO_2_; **(B)** ZrO_2_; **(C)** TiO_2_.ZrO_2_.

The X-ray diffraction patterns of the synthesized materials under different conditions calcined for various periods at 80°C and at 500°C are presented in Figures 1D, Figure 2, respectively. The nanostructure materials calcined at 80°C (Figure 1D) do not show the reflexions corresponding to any metal oxide (ZrO_2_, TiO_2_, or TiO_2_.ZrO_2_), indicating that at this temperature crystallization has not taken place. The pure compounds aged at 500°C for 1 and 20 days present the characteristic peaks of anatase for the TiO_2_ (JDPDS JCPDS84-1285), and the cubic phase of zirconia was formed for the ZrO_2_ compound (JCPDS27-0997). These materials synthesized on fCNTs kept the same crystal structure with an average crystallite size, calculated by the Sherrer formula (d = kλ/β cos θ), of 5 nm for TiO_2_/fCNT and 7.1 nm for the ZrO_2_/fCNT (Figures 2A,B). The mixed compound TiO_2_. ZrO_2_/fCNT aged at 500°C showed an amorphous diffraction pattern (Figures 2C–E), indicating either amorphousness or too small nanoparticles that do not show sharp peak signals in the XRD pattern. The latter suggests that Zr_4_^+^ could suppress or delay the crystallization of the ZrO_2_–TiO_2_ system, and the growth rate is inhibited by the presence of both metal oxides. Similar results have been reported in the literature for the TiO_2_–ZrO_2_ system calcined in the range 400–550°C for ZrO_2_ content over 50%; in our case, the ZrO_2_ content is 60%. Therefore this is in good agreement with the results reported by different authors (Liu et al., 2003; Wan et al., 2004; Kitiyanan et al., 2006).

The results from FTIR and XRD showed that the fCNT/TiO_2_.ZrO_2_ (36)500°C.20 material presented the best performance; therefore, this material was chosen for the subsequent studies.

#### 3.1.3 UV-Vis Spectroscopy

**FIGURE 3:**
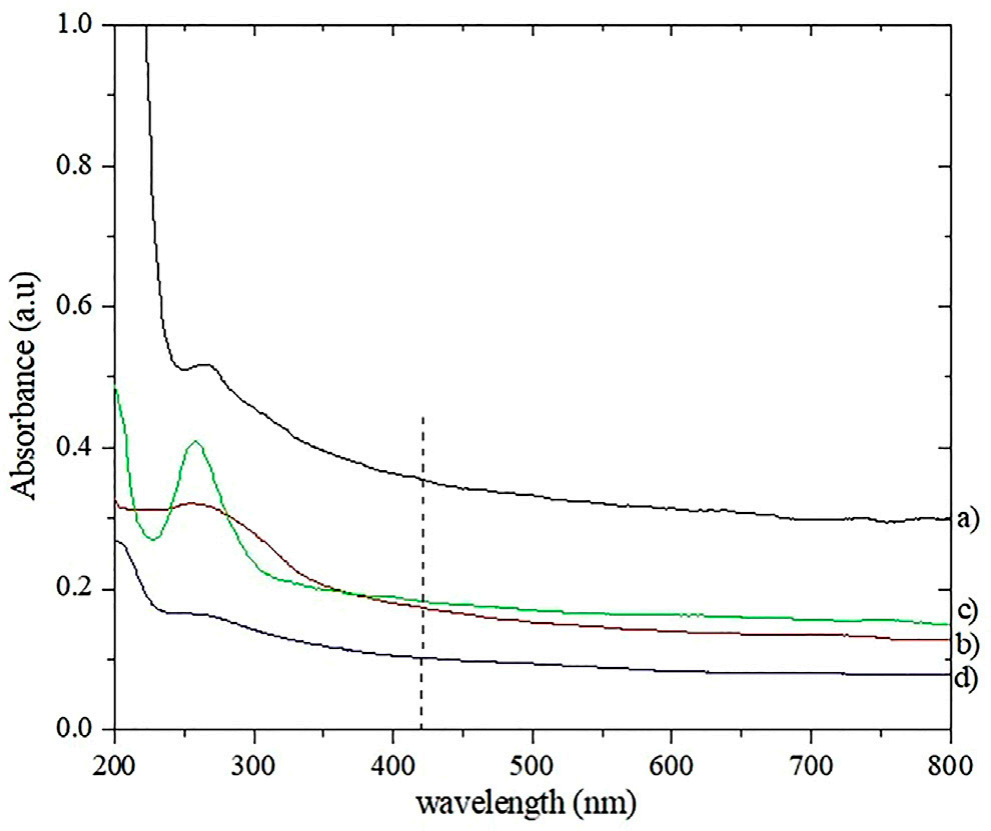
UV-visible spectra: **(A)** fCNT; **(B)** fCNT/TiO_2_(36)500°C.20; **(C)** fCNT/ZrO_2_(36)500°C.20; **(D)** fCNT/TiO_2_.ZrO_2_(36)500°C.20.

The UV-vis spectra of the nanostructure mixed oxide material fCNT/TiO_2_.ZrO_2_(36)500°C.20 is compared in Figure 3 with the single metal oxides (MO_2_) on carbon nanotubes: fCNT/TiO_2_ (36)500°C.20, fCNT/ZrO_2_ (36)500°C.20, and the fCNT. It can be observed that the mixed oxide nanostructured material fCNT/TiO_2_.ZrO_2_(36)500°C.20 presents a lower absorption at 258 nm than the fCNT/MO_2_ systems. This could be attributed to the very small particle size obtained for this material, as was observed by XRD. This agrees with the XRD patterns reported for the mixed TiO_2_-ZrO_2_ system reported by Kitiyanan et al. (2006).

#### 3.1.4 Transmission Electron Micrographs

**FIGURE 4:**
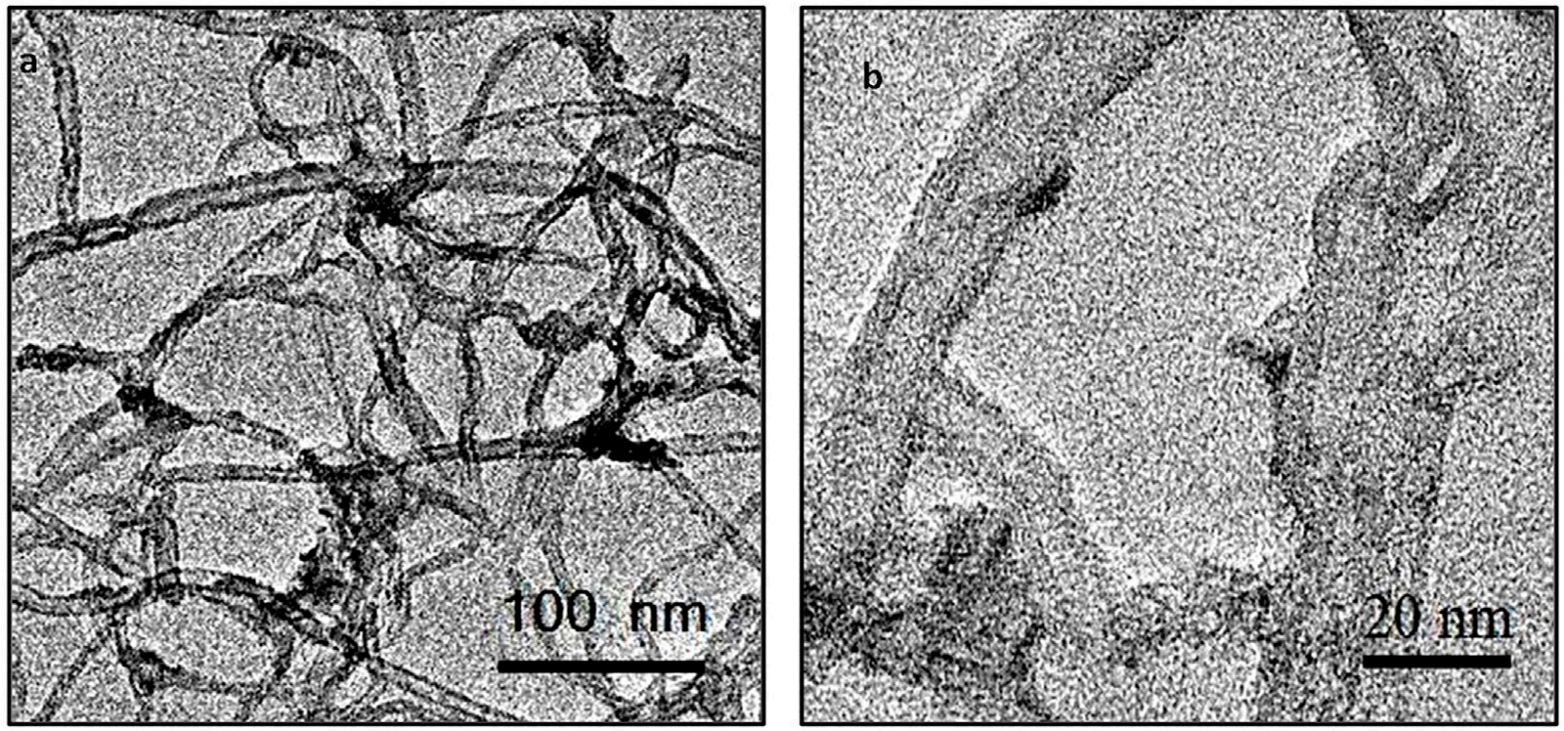
TEMs of fCNT/TiO_2_.ZrO_2_(36)500°C.1: **(A)** general view and **(B)** detailed view of NPs on fCNT walls.

**FIGURE 5:**
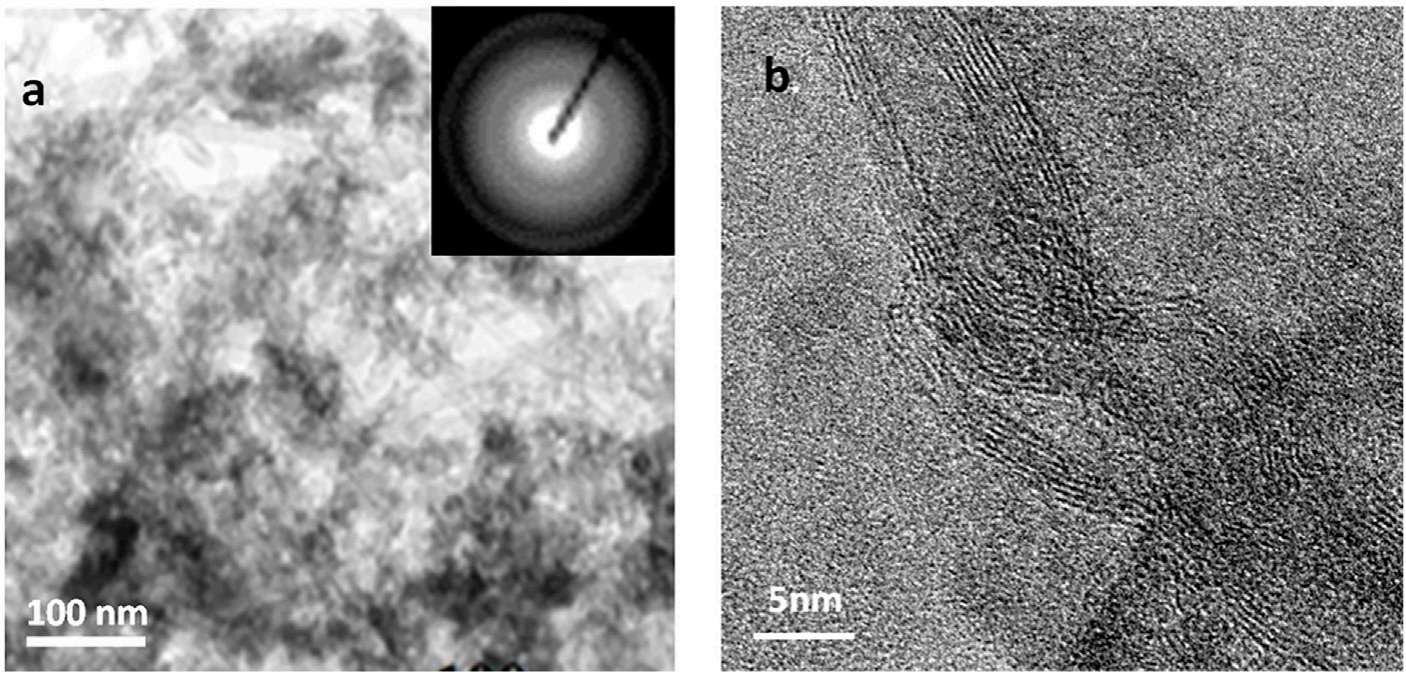
TEMs of fCNT/TiO_2_.ZrO_2_(36)500°C.20: **(A)** general view showing Nps covering fCNTs; inset: electron diffraction pattern indicating the amorphous character of the Nps; **(B)** HRTEM showing Nps on fCNT walls.

TEMs of the mixed oxide nanostructured materials calcined at 500°C and aged for 1 and 20 days (fCNT/TiO_2_-ZrO_2_ (36)500°C.1 and fCNT/TiO_2_.ZrO_2_ (36)500 °C.20 are shown in Figures 4, 5, respectively. It can be observed that after 1 day, very few particles were formed (Figure 4), consistent with the FTIR and XRD results. However, after 20 days of aging (Figure 5), a large number of nanoparticles are observed covering the fCNT walls. Figure 5A is a general view of the material after 20 days of aging, showing a large number of nanoparticles, and the inset show the electron diffraction pattern indicating the amorphous characteristic of these nanoparticles. Figure 5B is a high-resolution electron microscopy (HREM) image of the same material showing the distribution of the nanoparticles on the fCNT walls.

#### 3.1.5 Thermogravimetric Analysis

**FIGURE 6:**
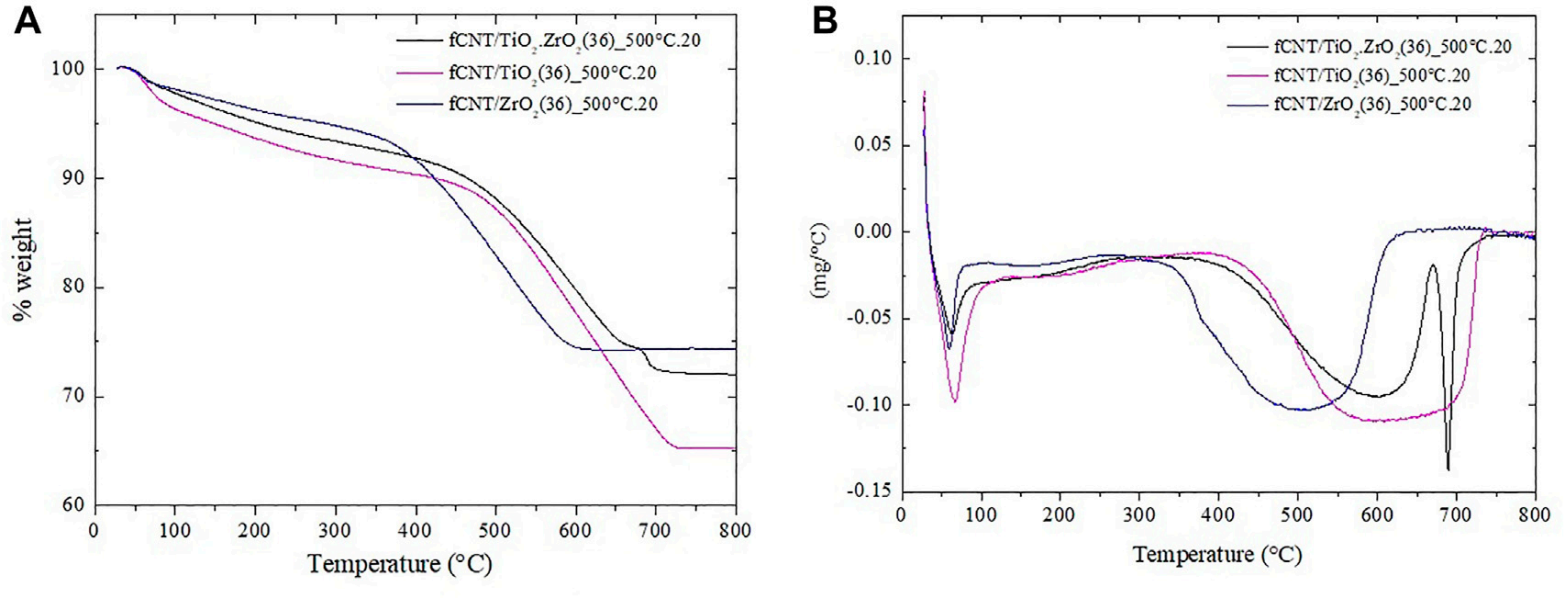
Thermograms of nanostructured materials fCNT/MO_2_(36)500°C.20: **(A)** TGA plot and **(B)** DTG plot.

The fCNT/TiO_2_.ZrO_2_ (36)500°C.20 material loses 28 w%. This represents an intermediate weight loss compared with the fCNT/TiO_2_(36)500°C.20 and fCNT/ZrO_2_ (36)500°C.20 materials (Figure 6). In total, four stages of weight loss are observed: the first between 33 and 100°C with a minimum in the DTG at 63.2°C associated with desorbed water; the second stage between 100 and 290°C corresponds to remained amorphous carbon; the third stage between 352 and 670°C represents the degradation and decomposition of fCNT; and the fourth stage from 670 to 739°C presents a sharp minimum in the DTG plot at 689°C, associated to the crystallization of TiZrO_4_. Zou and Lin (2004) reported minima values at 754, 712, and 606°C for TiO_2_/ZrO_2_ mixtures with ZrO_2_ molar contents of 25, 50, and 75%, respectively. The molar ratio of ZrO_2_ obtained in the present work should be near 60%, as was theoretically calculated; therefore the sharp minimum at 689°C would correspond to the crystallization of the ZrO_2_-TiO_2_ system with the formation of a new phase ZrTiO_4_ (Zou and Lin, 2004). The latter is in good agreement with the ZrO_2_–TiO_2_ phase diagram (Troitzsch and Ellis, 2004). Furthermore, the final decomposition temperature of the fCNTs could be between 640 and 740°C. Table 2 presents the different stages and the percentage of fCNTs with respect to the MO_2_, which is slightly lower than the theoretical value, especially for the fCNT/TiO_2_ (36)500°C.20 and fCNT/ZrO_2_ (36)500°C.20 materials. The minima values of the DTG curves for the fCNT/TiO_2_.ZrO_2_ (36)500°C.20 material are at intermediate positions compared with the fCNT/TiO_2_ (36)500 °C.20 and fCNT/ZrO_2_ (36)500°C.20 compounds.

**TABLE 2:** Thermogravimetric analysis.

	Temperature (°C)	Minimum DTG (°C)	Weight loss (%)	fCNT %
fCNT/TiO_2_.ZrO_2_ (36)500°C.20	33–100	63.20	2.20	25
	100–290	170.6	4.30	
	352–670	602.1	18.10	
	670–739	689.2	2.50	
fCNT/TiO_2_.ZrO_2_ (36)500°C.20	33–128	65	4.40	35
	128–337	210	4.10	
	370–740	–	25.40	
fCNT/TiO_2_.ZrO_2_ (36)500°C.20	33–97	58.0	1.80	28
	97–267	156.0	2.90	
	267–638	510.3	21.0	

#### 3.1.6 Surface Area

**FIGURE 7:**
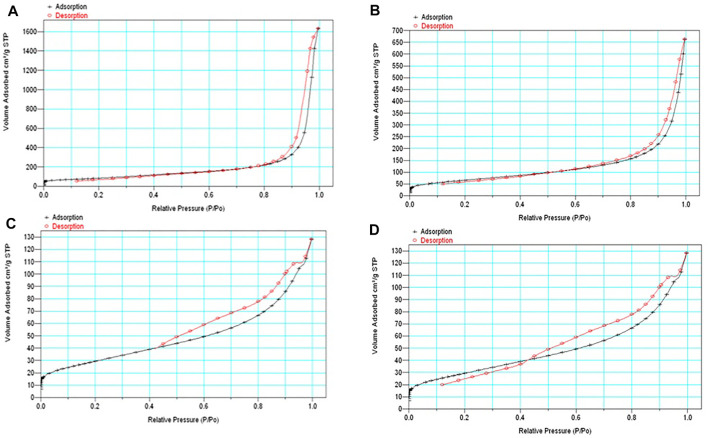
N_2_ adsorption–desorption isotherms: **(A)** fCNT, **(B)** fCNT/TiO_2_.ZrO_2_(36)500°C.20, **(C)** fCNT/TiO_2_(36)500°C.20, and **(D)** fCNT/ZrO_2_(36)500°C.20.

The surface area of the nanostructured systems fCNT/TiO_2_.ZrO_2_ (36)500°C.20 is lower than that of fCNT/TiO_2_ (36)500°C.20 and fCNT/ZrO_2_ (36)500°C.20 (Table 3 and Figure 7), attributed to the larger amount of TiO_2_.ZrO_2_ nanoparticles covering the fCNTs, therefore decreasing the surface area.

**TABLE 3:** Surface area BET of the nanostructured materials.

Material	BET area (m^2^/G)	Mass% of fCNTs relative to oxide by TGA
fCNT	298.40 ± 2,72	100
fCNT/TiO_2_.ZrO_2_ (36)500°C.20	81.54 ± 1,13	25
fCNT/TiO_2_ (36)500°C.20	147.02 ± 1,89	39
fCNT/ZrO_2_ (36)500°C.20	92.12 ± 1,03	28

#### 3.1.7 Electrochemical Characterization

**FIGURE 8:**
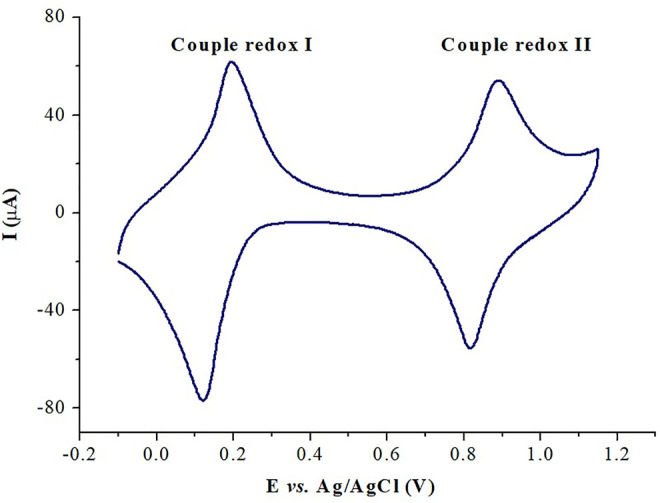
Cyclic voltammetry of PB-fCNT/TiO_2_.ZrO_2_(36)500°C.20/GC-modified electrodes in PBS, pH 3.0 at 40 mVs^-1^.

Cyclic voltammetry was used to evaluate the electrochemical behavior of the PB-fCNT/TiO_2_.ZrO_2_ (36)500°C.20 film on the GC electrode. Figure 8 shows the CV obtained for the PB-fCNT/TiO_2_.ZrO_2_ (36)500°C.20/GC electrode recorded at a scan rate of 40 mV s^−1^ in PBS 0.1 mol L^−1^ (pH 3) as a supporting electrolyte. The CV shows two well-defined redox couples, with potential E_1/2_ (E_1/2_= (E_pa_ + E_pc_)/2) for the redox couple I and II of 0.189 and 0.875 V (*vs*. Ag/AgCl), respectively; these values were independent of the scan rate. The redox couple I signal between 0.3 and −0.1 V is associated with high-spin redox reactions Fe(CN)_6_^3−⁄4−^ (the conversion of Prussian blue (PB) to Prussian white (PW) species, reaction 1), and the redox couple II signal between 0.7 and 1.1V corresponds to the reaction of low spin Fe^3+/2+^ (Prussian blue (PB) to Berlin green (BG) species, reaction 2). The electrochemical behavior described of the PB on the electrode PB-fCNT/TiO_2_.ZrO_2_ (36)500°C.20/GC agrees with reports described in the literature for PB Nps (Li et al., 2007; Adekunle and Ozoemena, 2010; Cantanhêde Silva et al., 2010; Kong et al., 2015).

$$Fe_{4}^{III}{\left[Fe^{II}{\left(CN\right)}_{6}\right]}_{3}+4e^{-}+4K^{+}⇄{\left[K_{4}Fe_{4}^{II}{\left(CN\right)}_{6}\right]}_{3}$$ (R1)

PB PW

$$Fe_{4}^{III}{\left[Fe^{II}{\left(CN\right)}_{6}\right]}_{3}+4A^{-}+⇄K_{4}Fe_{4}^{III}{\left[Fe^{III}{\left(CN\right)}_{6}A\right]}_{3}+3e^{-}$$ (R2)

PB, BG

For the PB-fCNT/TiO_2_.ZrO_2_ (36)500°C.20/GC-modified electrode, the voltammetry signals in the reduction zone show an Ipa/Ipc (peak anodic current /peak cathodic current) of 0.86, and the ∆Ep (the separation of the peak potential) is 0.221 V, while in the oxidation zone, the ratio Ipa/Ipc is 0.95, and ∆Ep is 0.151 V. These results suggest a quasi-reversible reaction process of PB on the surface of the modified electrode.

According to the Randles–Sevcik equation (Wang, 2000), Figure 9 shows a linear plot of Ipc and Ipa versus ν^1⁄2^, for the PB-fCNT/TiO_2_.ZrO_2_ (36)500°C.20/GC electrode, revealing a diffusion-controlled process, which we have related to the slow diffusion of potassium ions in the lattice of the film on the electrode (Karyakin, 2001).

**FIGURE 9:**
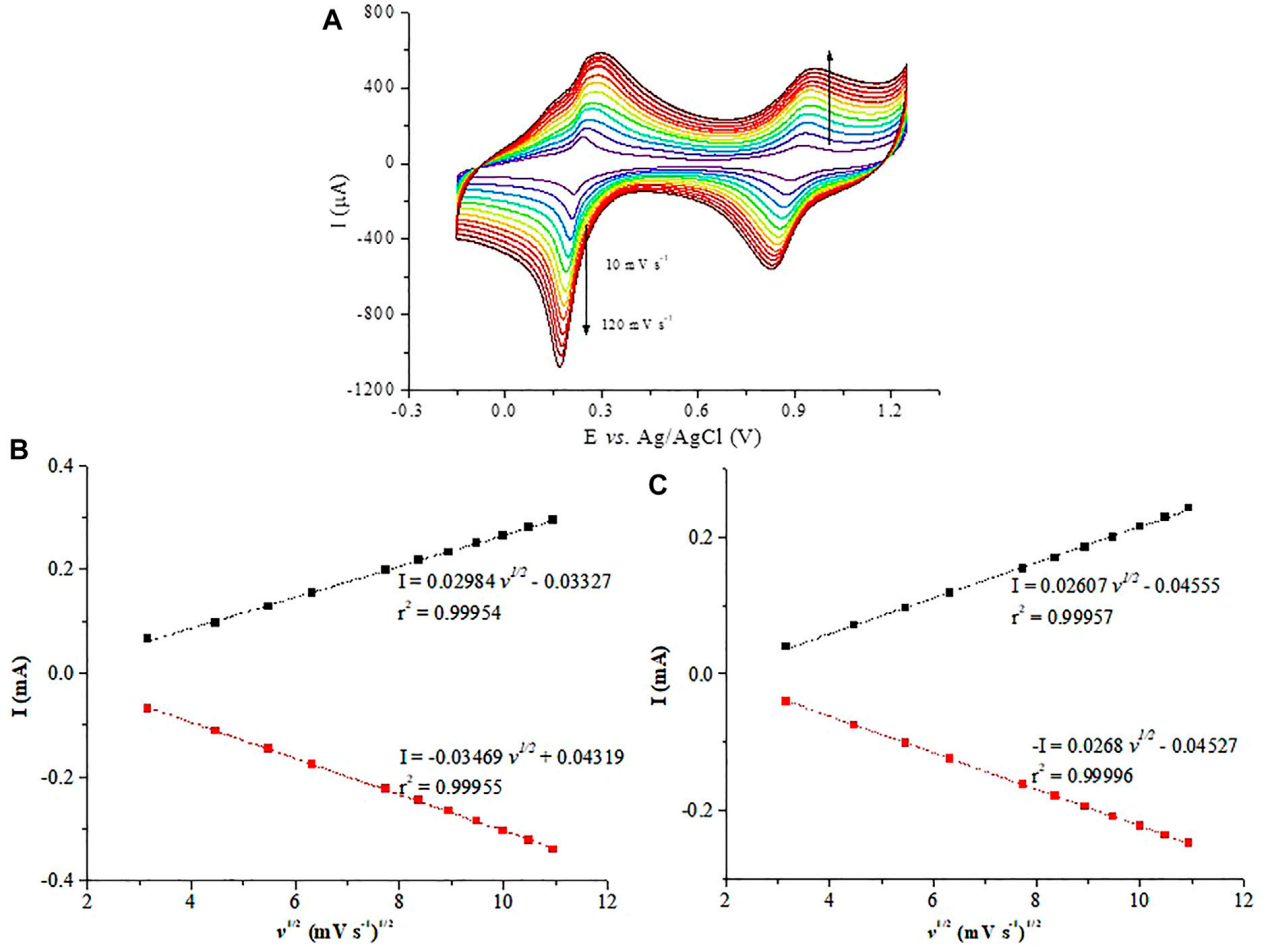
**(A)** Cyclic voltammetry at PB-fCNT/TiO_2_.ZrO_2_(36)500°C.20/GC-modified electrode in PBS at pH 3.0 at different scan rates; **(B)** PB reduction; **(C)** PB oxidation peaks current vs. the square root of the scan rate, I vs. (v)1/2.

The surface concentration ( $\Gamma_{c}$ ) of PB on the modified electrode was evaluated from the slope I_p_/A versus ν, according to the following equation (Tang et al., 1994; Du et al., 2010):

$$Ip=\frac{n^{2}F^{2}Av\Gamma c}{4RT}$$ (4)

where Ip is the current (A), A is the surface area of the electrode (cm ^−2^), ν is the potential scan rate (Vs^−1^), n is the number of electrons, R is the gas constant (J mol^−1^ K^−1^), T the temperature (K), and F the Faraday constant (C mol^−1^). The average value of Γc or the redox peaks was 1.07 × 10^−8^ mol cm^−2^ (for n = 4 and ν < 500 mVs^−1^). Γc for electrode PB-fCNT/TiO_2_.ZrO_2_ (36)500°C.20/GC is one order of greater magnitude than the prepared electrodes with metal oxides individually; PB-fCNT/ZrO_2_ (36)500°C.20/GC and PB-fCNT/TiO_2_ (36)500 °C.20/GC are reported in the literature (Guerrero et al., 2019; Jerez-Masaquiza et al., 2020), Table 4. This result suggests that the oxides ZrO_2_ and TiO_2_ can act altogether in order to control PB nucleation either on the nanotubes, acting as a seed for PB film growth or as a “coating agent”, resulting in a larger electroactive surface area for the PB growth on the electrode surface.

**TABLE 4:** Comparison of the obtained electrochemical parameters for the PB-fCNT/TiO_2_.ZrO_2_ (36)500°C.20/GC electrode with the prepared electrodes under the same conditions with the individual oxides in their structure (Li et al., 2007; Rathee et al., 2021).

Parameter/Electrode	PB-fCNT/GC	PB-fCNT/TiO_2_.ZrO_2_ (36)500°C.20/GC	PB-fCNT/ZrO_2_ (36)500°C.20/GC	PB-fCNT/TiO_2_ (36)500°C.20/GC
E_1/2_ (reduction/oxidation) /mV	0.22/0.88	0.23/0.90	0.23/0.90	0.23/0.87
Ipa/Ipc (reduction/oxidation)	0.86/1.13	0.80/0.90	0.79/1.6	0.88/1.44
Γ_c_ (mol cm^−2^)	3.98 × 10^−10^	1.07 × 10^−8^	1.2 × 10^−9^	4.72 × 10^−9^
Capacitance (mF cm^−2^)	2.37	2.96	1.51	1.48
k_s_ (s^−1^)	2.7 × 10^−2^	8.47 × 10^−5^	6.04 × 10^−3^	8.70 × 10^−4^

The electrochemical capacitance, C_dl_, of the modified electrode was studied by CV, and this was carried out in PBS (pH 3.0) and potentials of 0.20–0.60 V (Haghighi et al., 2010). The following equation describes that the background current is a function of the scan rate:

$$j=I_{c}/A=C_{dl}v$$ (5)

where A is the effective surface area, ν is the scan rate, and C_dl_ is the electrochemical double-layer capacitance. Plot I_c_/A versus ν presented a straight line where the C_dl_ of the modified electrode was obtained from the slope (data not shown). The C_dl_ obtained for PB-fCNT/TiO_2_.ZrO_2_ (36)500°C.20/GC was 2.96 mF cm^−2^; this is a higher value than that reported for electrodes prepared under the same conditions with the individual oxides (MO_2_), Table 4. This indicates that the PB-fCNT/TiO_2_.ZrO_2_(36)500°C.20/GC electrode has a larger specific area than individually modified electrodes with each oxide, which agrees with the results obtained by calculating the BET surface area.

Based on Laviron's theory (Zhang et al., 2003a; Lin et al., 2003), the electron transfer rate constant (k_s_) was determined for the modified electrode in PBS (pH 3), measuring the variations of the anodic and cathodic potential peaks at different scan rates, when ΔEp was superior to 100/n mV. Assuming *n* = 4, the calculated value for ks was 5.47 × 10^−5^ s^−1^, lower value than that reported for electrodes prepared under the same conditions with the individual oxides in their structure, Table 4, which suggests that the PB-fCNT/TiO_2_ (36)500°C.20/GC electrode favors the electronic transfer on its surface.

**FIGURE 10:**
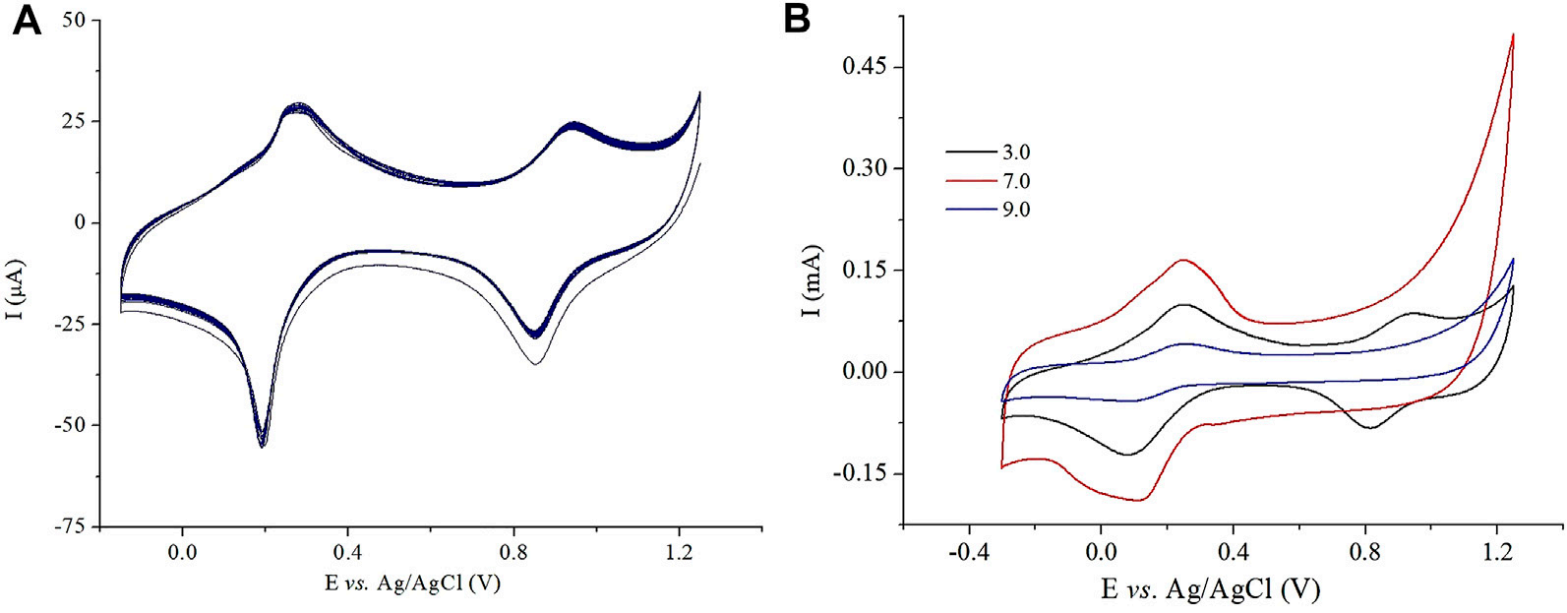
Cyclic voltammetry at the PB-fCNT/TiO_2_.ZrO_2_(36)500°C.20/GC-modified electrode in **(A)** PBS (pH 3), 40 mV/s, and 90 cycles; **(B)** PBS, pH 3.0, 7.0, and 9.0.

The stability of the modified electrode PB-fCNT/TiO_2_.ZrO_2_ (36)500°C.20/GC was tested by measuring the decrease of the voltammetry currents during the potential cycles. The electrode showed no decrease in the current in the supporting electrolyte (Figure 10A), when subjected to 90 cycles of potential from –0.15 to +1.25 V at a scan rate of 40 mV/s. We have associated the good stability of the electrode to the fact that the nanomaterial fCNT/TiO_2_.ZrO_2_ has good electrical conductivity, where the carbon atoms of the fCNT, as electron donors, and the CN of PB, as electron acceptors, present efficient π–π interaction (Shim et al., 2002; Zhang et al., 2003b; Lin et al., 2003); also, the iron cations in PB successfully interact with the carboxyl anions of fCNTs (Diao et al., 2002), thus generating an electrochemically efficient composite.

In addition, the stability of the modified electrode PB-fCNT/TiO_2_.ZrO_2_ (36)500°C.20/GC was evaluated by cyclic voltammetry at different pH values. Figure 10B shows the current intensity between the third and the first cycle at pH values ranging between 3.0.7.0 and 9.0. At pH 3.00, the electrode showed the best stability, with a slight decrease in current during cycles. At pH 9, a rapid decrease in current intensity is observed, which may be related to the formation of Fe(OH)_3_ and the consequent decomposition of PB (Stilwell et al., 1992); at pH 7, well-defined PB to PW transformation peaks can be observed but the PB to BG transformation peaks are distorted until they disappear. As these results show, a very high stability of the electrode in acid solution was obtained, where pH 3.0 is considered as the most suitable value for the supporting electrolyte and the application of the PB-fCNT/TiO_2_.ZrO_2_ (36)500°C.20/GC electrode as a sensor of H_2_O_2_; therefore, this is the pH that was maintained for subsequent studies.

### 3.2 Electrocatalytic Behavior

Reduction of H_2_O_2_ at a PB-modified electrode surface has been shown to follow the classical two-electron reduction (reaction 3) (Sitnikova et al., 2014; Noël et al., 2016):

$$H_{2}O_{2}+2e^{-}↔2HO^{-}$$ (R3)

where the loss of stability of the electrode is generally associated with the progressive loss of catalytic activity over time, which is attributed to the basification of the environment near the electrode surface by the production of OH^−^ ions in the reduction of H_2_O_2_ since PB is known to react with HO-according to the reaction (Montes et al., 2016) (Ricci et al., 2003):

$$Fe_{4}^{III}{\left[Fe^{II}{\left(CN\right)}_{6}\right]}_{3}+12OH^{-}\to 4Fe{\left(OH\right)}_{3}+3{\left[Fe^{II}{\left(CN\right)}_{6}\right]}^{4-}$$ (R4)

For the case of the PB-fCNT/TiO_2_.ZrO_2_ (36)500°C.20 material, the obtained results for the Z potential (not shown) indicated that its surface is negatively charged, which can contribute to the repulsion of surface OH^−^ ions and thus increases the stability of the PB-fCNT/TiO_2_.ZrO_2_ film on the sensor surface.

**FIGURE 11:**
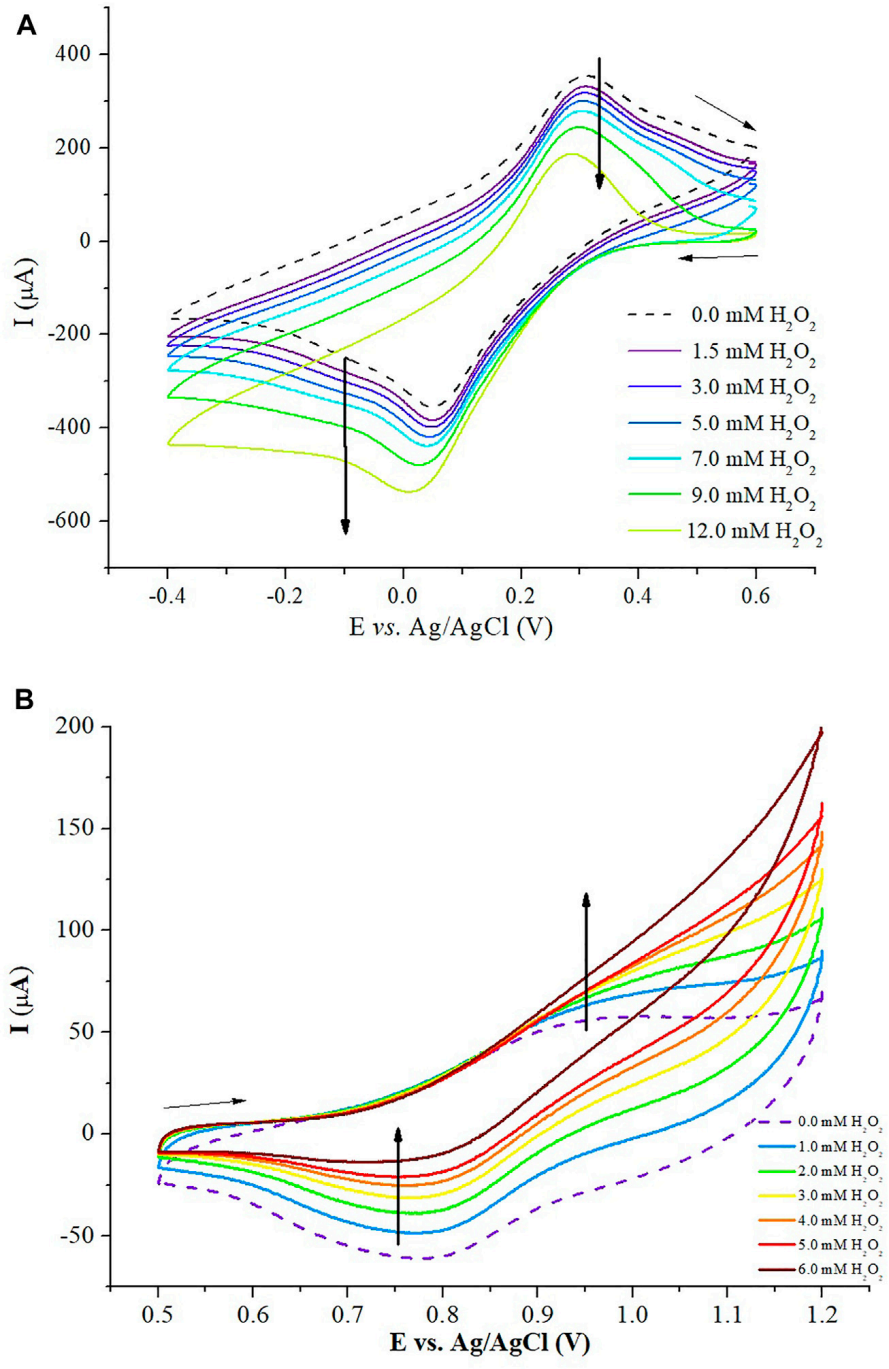
**(A)** Cyclic voltammetry of H_2_O_2_ at the PB-fCNT/TiO_2_.ZrO_2_(36)500°C.20/GC-modified electrode (reduction zone) in 0.1 mol L^-1^ PBS (pH. 3.0) at 40 mV s^-1^. **(B)** Cyclic voltammetry of H_2_O_2_ at the PB-fCNT/TiO_2_.ZrO_2_(36)500°C.20/GC-modified electrode (oxidation zone) in 0.1mol L^−1^ PBS (pH. 3.0) at 40 mV s^-1^.

To evaluate the catalytic activity of the electrode PB-fCNT/TiO_2_.ZrO_2_ (36)500°C.20/GC for H_2_O_2_, cyclic voltammograms were taken in the presence and absence of this analyte (Figure 11). In the presence of H_2_O_2_, both in the zone of conversion from PB to PW, reaction 1 (Figure 11A), and in the zone of conversion from PB to BG, reaction 2 (Figure 11B), it showed a decrease in reverse redox currents and an increase in forward redox currents, demonstrating that electrocatalytic reduction and oxidation, respectively, of H_2_O_2_ occurred on PB-fCNT/TiO_2_.ZrO_2_ (36)500°C.20/GC–modified electrode. The redox peaks in the voltammogram of Figure 11B show electrocatalysis toward the oxidation of H_2_O_2_ (R6), while the redox peaks in Figure 11A show the electrocatalysis of the reduction of H_2_O_2_ (R5).

The reduction reaction is as follows:

$$K_{4}Fe_{4}^{II}{\left[Fe^{II}{\left(CN\right)}_{6}\right]}_{3}+2H_{2}O_{2}⇄Fe_{4}^{III}{\left[Fe^{II}{\left(CN\right)}_{6}\right]}_{3}+4OH^{-}+4K^{+}$$ (R5)

The oxidation reaction is as follows:

$$2Fe_{4}^{III}{\left[Fe^{III}{\left(CN\right)}_{6}\right]}_{3}+A_{3}+3H_{2}O_{2}⇄2Fe_{4}^{III}{\left[Fe^{II}{\left(CN\right)}_{6}\right]}_{3}+3O_{2}+6A^{-}+6H^{+}$$ (R6)

However, according to the voltammograms in Figure 11A, on the PB-fCNT/TiO_2_.ZrO_2_ (36)500°C.20/GC electrode, a better response was obtained for the electroreduction of H_2_O_2_ compared with the electrooxidation obtained in the voltammogram with the increase in peroxide concentration Figure 11B.

**FIGURE 12:**
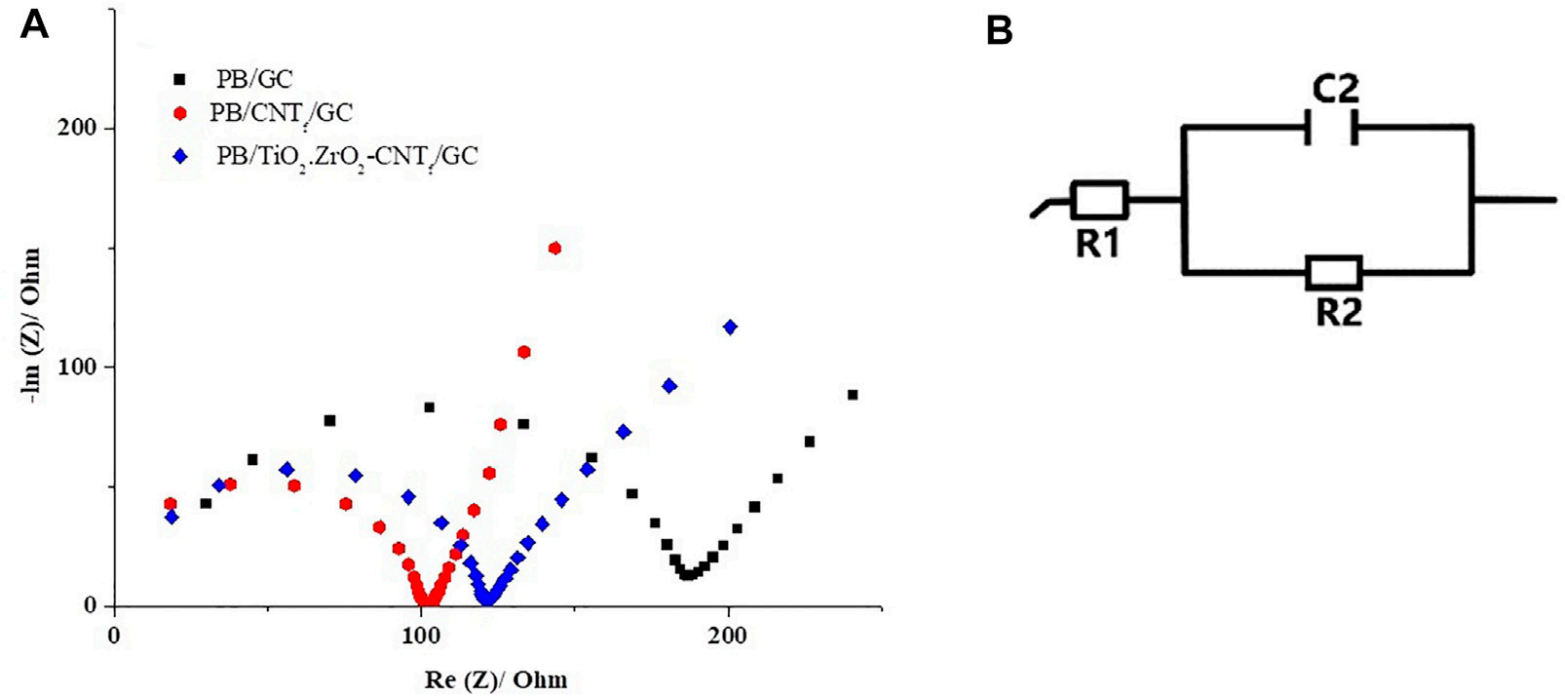
**(A)** Nyquist plots: PB/GC, PB/fCNT/GC, and PB/TiO_2_.ZrO_2_- fCNT/GC-modified electrodes in 7.5 mmol L H_2_O_2_ in 0.1 mol 1.1 mol/L. PBS (pH 3.0). **(B)** Equivalent circuit of the three electrodes.

Electrochemical impedance spectra (EIS), from 40 KHz to 5 MHz, 0.01 Hz to 2 MHz, and 0.1 Hz to 5 MHz for PB/GC, PB/_f_CNT/GC, and PB/TiO_2_.ZrO_2_-fCNT/GC electrodes, respectively, and an applied sinusoidal perturbation of 10 mV, in the presence of H_2_O_2_, were recorded. Figure 12 shows the impedance spectra at 0.10 V (reduction of H_2_O_2_ potential) for PB/GC, PB/fCNT/GC, and PB/TiO_2_.ZrO_2_-fCNT/GC electrodes. At the PB/GC electrode (black curve), charge transfer resistance (Rct) at a high frequency is higher than for the other electrodes. Immobilization of PB/TiO_2_.ZrO_2_-fCNT films on the GC (red curve) produced the smallest semicircle, which demonstrated that PB/TiO_2_.ZrO_2_-fCNT/GC-modified electrodes have a higher Rct value than PB/GC and PB/fCNT/GC (blue curve); this implies that TiO_2_.ZrO_2_ nanoparticles play an important role in providing the conducting bridges for the charge transfer of H_2_O_2_ at PB/TiO_2_.ZrO_2_-fCNT/GC-modified electrode, in agreement with the literature (Yang et al., 2016).

To evaluate the rate constant, K_c_, for the catalytic reaction, the current signals were used on the electrode PB-fCNT/TiO_2_.ZrO_2_ (36)500°C.20/GC in the presence (I_c_) and in the absence of H_2_O_2_, (I_L_), according to the following equation (Galus et al., 1976):

$$\frac{I_{c}}{I_{L}}={\left(K_{c}C\pi \right)}^{1/2t1/2}$$ (6)

where K_c_, C, and t are the rate constant of the catalytic chemical reaction (cm^3^ mol^−1^ s^−1^), concentration of H_2_O_2_ in the bulk of the solution (mol cm^−3^), and the time (s), respectively. From the slope of the plot of I_c_/I_L_ versus t^−1/2^ (not shown), the K_c_ value for a fixed concentration of H_2_O_2_ was obtained. The average value of K_c_ (*n* = 3) in the concentration range of 150 at 1.028 μmol L^−1^ was 11.52 × 10^4^ cm^3^ mol^−1^ s^−1^, which is two orders of magnitude smaller than the values of ×10.7310^6^ and 9.84 × 10^8^ cm^3^ mol^−1^s^−1^ previously reported for the electrodes PB-fCNT/ZrO_2_(36)500°C.20/GC and PB-fCNT/TiO_2_ (36)500°C.20/GC, respectively (Guerrero et al., 2019; Jerez-Masaquiza et al., 2020).

### 3.3 Electroanalytic Behavior

**FIGURE 13:**
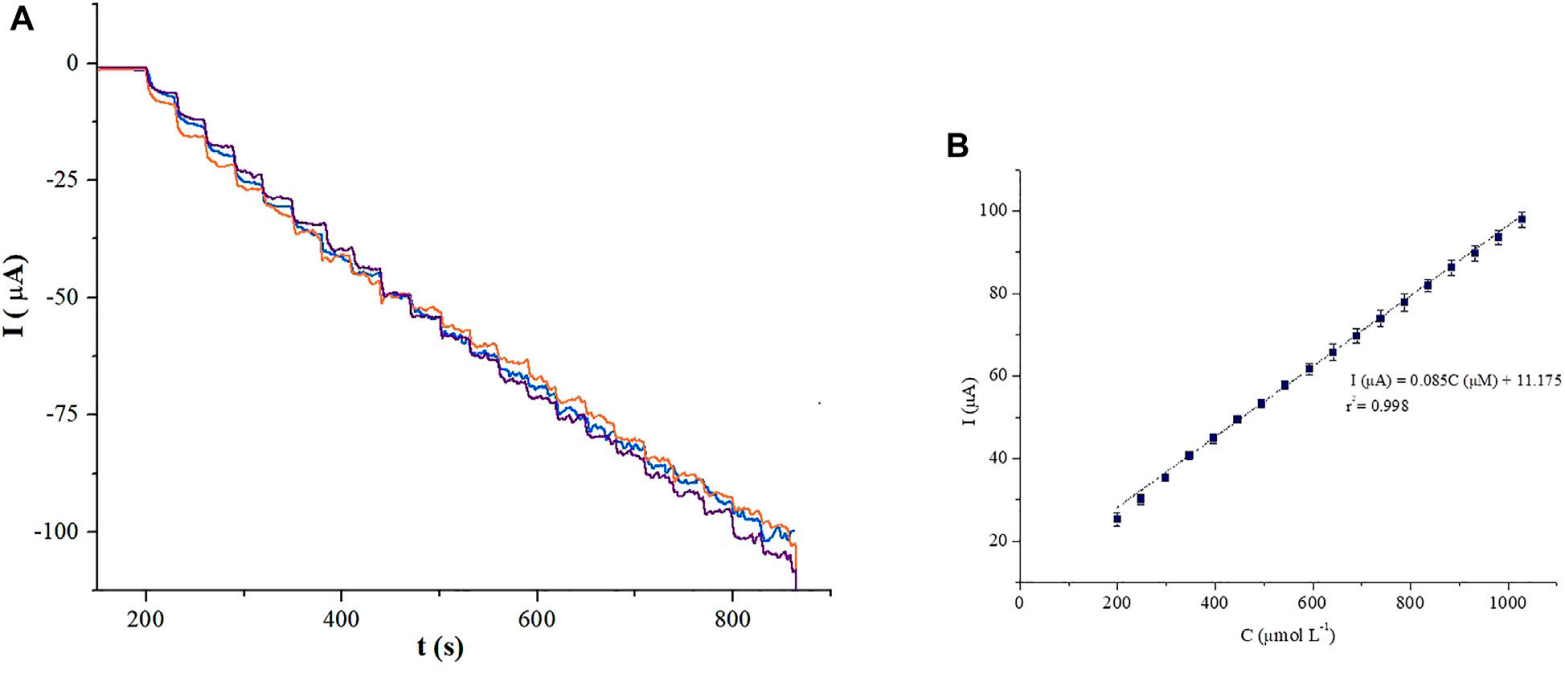
Chronoamperometric detection of H_2_O_2_ at the PB-fCNT/TiO_2_.ZrO_2_(36)500°C.20/GC electrode, in PBS (pH 3) solution **(A)** and its **(B)** calibration plot. Working potential of −0.03 V vs. Ag/AgCl.

Figure 13A shows the chronoamperometric response on the PB-fCNT/TiO_2_.ZrO_2_ (36)500°C.20/GC electrode, after adding 0.050 mmol/L of H_2_O_2_ in PBS (pH 3) at –0.03 V. A linear H_2_O_2_ signal on the PB-fCNT/TiO_2_.ZrO_2_ (36)500°C.20/GC electrode for a concentration range from 1,000 μmol L^−1^ was obtained, Figure 13B, with a quantification limit (QL) of 59.78 μmol L^−1^ and the detection limit (DL) of 17.93 μmol L^−1^. The PB-fCNT/TiO_2_.ZrO_2_ (36)500°C.20/GC electrode amplified the hydrogen peroxide current ∼100 times compared with the electrodes PB-fCNT/TiO_2_ (36)500°C.20/GC (Guerrero et al., 2019), which could be attributed to the good synergy of the nano oxides together on electrocatalysis to reduce hydrogen peroxide. The PB encapsulation in the internal channels of fCNTs compared to PB on the external surface improves electrochemical properties (Yagi et al., 2004; Chen et al., 2007); this is why it is suggested that the electronic interaction between the d-orbital of Fe electron in PB and the inner surface of the modified fCNT improves the electrocatalytic performance of the PB complex on this electrode surface. Therefore, the huge increase of the current densities in the presence of H_2_O_2_ on the PB-fCNT/TiO_2_.ZrO_2_ (36)500°C.20/GC electrode could also be attributed to the supramolecular electronic interactions between PB and the fCNT. On the other hand, according to the previous results (Guerrero et al., 2019), the PB-fCNTs/GC electrode showed an unstable behavior at the applied potentials for H_2_O_2_ detection. The comparison between the fCNT/TiO_2_.ZrO_2_ (36)500°C.20/GC electrode with the one previously reported for the PB-fCNT/ZrO_2_ (36)500°C.20/GC electrode (Jerez-Masaquiza et al., 2020) has not been carried out since the latter, besides working on a different redox zone, presented issues for its optimal preparation. Therefore, the modified electrode fCNT/TiO_2_.ZrO_2_ (36)500°C.20/GC was considered the best electrode for the evaluation of the of H_2_O_2_ detection in operability terms.

To assess the reproducibility response of the modified electrode PB-fCNT/TiO_2_.ZrO_2_ (36)500°C.20/GC, 10 (Lv et al., 2020) electrodes were made from the same GC electrode and independently showed acceptable reproducibility in the current value with relative standard deviation of 5.39% for 100 μmol L^−1^ H_2_O_2_. When the modified electrodes were not in use, they were stored in PBS (pH 3.0) at 4°C. In order to examine long-term storage stabilities, the voltammetric responses of the modified electrodes in 100 μmol L^−1^ H_2_O_2_ were monitored in regarding to storage time, every 5 days. After a storage period of 30 days, the PB-fCNT/TiO_2_.ZrO_2_ (36)500°C.20/GC electrode still retained the 78% of its initial current response to H_2_O_2_, indicating that the electrode has good stability and reproducibility.

**FIGURE 14:**
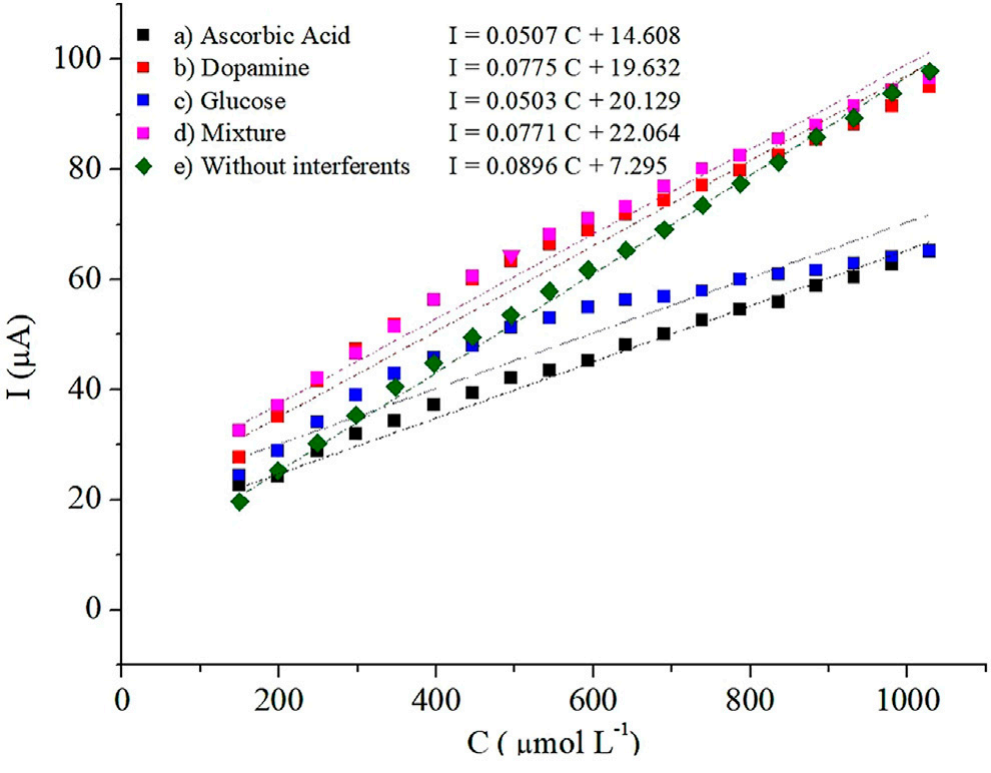
Calibration plots of H_2_O_2_ at the PB-fCNT/TiO_2_.ZrO_2_(36)500°C.20/GC electrode in PBS, pH 3 **(A)** with 10 mM of ascorbic acid; **(B)** 10 mM of dopamine; **(C)**10 mM of glucose; (D) 10 mM mixture of ascorbic acid, dopamine, and glucose; and (E) without interferents.

When a sensor is used for the determination of H_2_O_2_ in samples such as whey milk, the anti-interference capacity is an important parameter because coexisting species such as glucose (Glu), ascorbic acid (AA), and dopamine (DA) can interfere with the determination of H_2_O_2_. Figure 14 shows the calibration graphic for the electrocatalytic reduction of H_2_O_2_ by chronoamperometry at −0.03 V on the PB-fCNT/TiO_2_.ZrO_2_ (36)500°C.20/GC electrode in the presence of interfering species, including glucose (Glu), ascorbic acid (AA), dopamine (DA), and a mixture of them. The addition of individual species affected the electrode sensitivity in the following order: AA> Glu >DA; however, when all interferents are present in the solution, the sensitivity was not considerably affected.

Due to these results, the modified electrode PB-fCNT/TiO_2_.ZrO_2_ (36)500°C.20/GC was applied in the detection of hydrogen peroxide in whey milk samples. The accuracy of the developed analytical method was evaluated by calculating the recovery percentage (R%) in whey milk samples spiked with a known concentration of H_2_O_2_. Table 5 shows that the values for R% range between 94 and 104%; these values agree with a good accuracy of the method.

**TABLE 5:** Recovery data for H_2_O_2_ in spiked milk serum samples over a PB-fCNT/TiO_2_.ZrO_2_ (36)500°C.20/GC sensor.

Sample	Spiked amount (μmol L^−1^)	Measured amount (*n* = 3) (μmol L^−1^)	Recovery (%)
Milk serum	250	282.21 ± 12.12	93.12
	550	561.92 ± 6.93	100.08

Table 6 shows a comparison between the PB-fCNT/TiO_2_.ZrO_2_ (36)500°C.20/GC electrode and other electrodes reported in the literature. Table shows that our results are in agreement with other reports, suggesting that this electrode coating can be used to sense H_2_O_2_.

**TABLE 6:** Comparison of the results obtained at modified electrodes with other electrodes reported in the literature.

Modified electrode	Detection limit (mM)	Detection potential (V)	References
PB-fCNT/TiO_2_.ZrO_2_ (36)500°C.20/GC	1.79 × 10^−2^	−0.03	This work
Paper-based electrochemical sensing platform	4.0	0.00	Kaumal et al. (2021)
Hematite (α-Fe_2_O_3_) nanoarrays on fluorine-doped SnO_2_ glass (FTO)	2.0 × 10^−2^	−1.50	Lv et al. (2022)
Au/stainless-steel	3.97 × 10^−2^	−0.60	Huo et al. (2022)
Hybrid material–based polyaniline, dialdyhayed carboxmethyl cellouse, and in the presence of ZnO nanoparticles abbreviated	450	—	Althomali et al. (2022)
Silver nanoparticles (Ag NPs) entrenched in a silicate matrix (APS(SG))	2.5 × 10^−2^	−0.40	Maduraiveeran et al. (2018)
Gold nanocubes embedded biocompatible hybrid hydrogels	15	−0.15	Manickam et al. (2020)

## 4 Conclusion

An electrochemical sensor for H_2_O_2_ detection, PB-fCNT/TiO_2_.ZrO_2_ (36)500°C.20/GC, was efficiently designed with fCNT/TiO_2_.ZrO_2_–modified GC electrode. TiO_2_.ZrO_2_ nanoparticles on fCNTs were successfully prepared. From the XRD, DTG, and TEM/electron diffraction analysis, we could see that amorphous nanoparticles of TiO_2_.ZrO_2_ were formed on the fCNT walls, where Zr^4+^ suppresses or delays the crystallization of the ZrO_2_-TiO_2_ system. The calculated value for the electron transfer rate constant (k_s_) was lower than that reported for electrodes prepared under the same conditions with the individual oxides in their structure, which suggests that the PB-fCNT/TiO_2_.ZrO_2_ (36)500°C.20/GC electrode favors the electronic transfer at the PB–fCNT/GC electrode. Furthermore, the PB-fCNT/TiO_2_.ZrO_2_ layer exhibits good compatibility and affinity to the PB layer. The applicability of the sensor for detection of the hydrogen peroxide electrode was demonstrated in whey milk samples. Based on these advantages, the fabricated sensor exhibits good electrochemical sensibility, reversibility, and excellent linear relationship; nonetheless, the detection limit of the electrode can be improved. Finally, the fCNT/TiO_2_.ZrO_2_ layer can be used in the development of enzyme-based biosensors, which is the basis for future studies in our laboratory.

## Data Availability

The raw data supporting the conclusions of this article will be made available by the authors, without undue reservation.
